# Mental Health Among Lebanese Refugees in Syria

**DOI:** 10.1001/jamanetworkopen.2025.52793

**Published:** 2026-01-07

**Authors:** Ahmad Al-Bitar, Israa Tellawi, Omran Janoud, Kinda Al Issa, Haya Deeb, Hassan Fawaz, Youssef Latifeh

**Affiliations:** 1 Faculty of Medicine, Damascus University, Damascus, Syrian Arab Republic; 2 Faculty of Psychology, Damascus University, Damascus, Syrian Arab Republic; 3 Department of Psychiatry, Faculty of Medicine, Damascus University, Damascus, Syrian Arab Republic

## Abstract

This cross-sectional study assesses anxiety, depression, and sleep disturbance among a population of Lebanese refugees in Syria.

## Introduction

The October 2024 escalation of the conflict between Israel and Lebanon displaced many civilians in southern Lebanon, with some seeking refuge in neighboring Syria, a setting of prolonged instability. Refugees may face compounding premigration trauma and postdisplacement adversity,^1,2^ yet mental health among Lebanese refugees in Syria has not been quantified. We assessed anxiety, depression, and sleep disturbance in this population.

## Methods

This cross-sectional study was approved by the ethics committee of Damascus University, and all participants provided written informed consent. The study adhered to the STROBE reporting guideline.

We conducted a survey among Lebanese adults (aged ≥18 years) who had been displaced to Syria and resided in Damascus after the October 2024 escalation. Surveys were conducted from October 1 to November 15, 2024. Using Arabic-validated versions, we measured anxiety via the 7-item Generalized Anxiety Disorder scale, depression via the 9-item Patient Health Questionnaire, insomnia via the Insomnia Severity Index, and sleep quality via the Pittsburgh Sleep Quality Index (PSQI). We summarized sample characteristics and outcomes and used χ^2^ tests and 1-way analysis of variance for bivariate comparisons. Multivariable logistic regression models estimated adjusted odds ratios (AORs) and 95% CIs for dichotomized outcomes, adjusting for sex, age, educational level, marital status, employment, current housing, and war-related exposures. Two-sided *P* < .05 indicated statistical significance. Analyses were conducted using SPSS, version 26 (SPSS Inc). Detailed methodological information is provided in the eMethods in Supplement 1.

## Results

Among 305 participants (mean [SD] age, 36.3 [14.5] years; 170 [55.7%] female, 135 [44.3%] male), 48.9% were unemployed, 89.5% lived in hazardous or destroyed housing, and 59.0% reported smoking (Table 1). Current housing was associated with anxiety (χ^2^_6_ = 22.59; *P* = .001), depression (χ^2^_6_ = 25.41; *P* = .001), and insomnia (χ^2^_6_ = 15.29; *P* = .02), with lower anxiety and insomnia among those in private housing and the highest distress among those living with relatives. Participants with an injured close relative had higher PSQI scores than those without (mean [SD], 11.82 [5.86]; *P* = .04), and those whose homes were destroyed had the highest PSQI scores (mean [SD], 12.47 [5.46]; *P* = .02). Family injury status was associated with depression (χ^2^_8_ = 21.02; *P* = .007), with the highest prevalence of severe depression among those reporting an injured distant relative.

**Table 1.** Baseline Participant Characteristics

Characteristic	Value (N = 305)
Age, mean (SD), y	36.3 (14.5)
Sex, No. (%)	
Female	170 (55.7)
Male	135 (44.3)
Educational level, No. (%)	
Secondary school or less	66 (21.6)
Completed secondary school	115 (37.7)
University degree or higher	124 (40.7)
Work status, No. (%)	
Not working	149 (48.9)
Working	156 (51.1)
Marital status, No. (%)	
Single	112 (36.7)
Married	172 (56.4)
Divorced or widowed	21 (6.9)
Current smoker, No. (%)	180 (59.0)
Displacement-related factors, No. (%)	
Residency	
Private house	71 (23.3)
Shelter	66 (21.6)
With relatives	168 (55.1)
Housing status	
Safe	32 (10.5)
Dangerous or destroyed	273 (89.5)
Relative injured or killed	
No	64 (21.0)
Yes	241 (79.0)
Mental health	
Clinically significant anxiety (GAD-7 ≥10)	216 (70.8)
Clinically significant depression (PHQ-9 ≥10)	227 (74.4)
Clinically significant insomnia (ISI ≥15)	141 (46.2)

Abbreviations: GAD-7, 7-item Generalized Anxiety Disorder scale; ISI, Insomnia Severity Index; PHQ-9, 9-item Patient Health Questionnaire.

In adjusted models (Table 2), compared with unemployed participants, office workers had higher odds of moderate to severe anxiety (AOR, 2.75; 95% CI, 1.25-6.04), clinically meaningful insomnia (AOR, 2.98; 95% CI, 1.54-5.76), and moderate to severe depression (AOR, 3.14; 95% CI, 1.33-7.42). Living with relatives (vs private housing) was associated with higher odds of moderate to severe depression (AOR, 2.31; 95% CI, 1.09-4.89) and clinically meaningful insomnia (AOR, 2.14; 95% CI, 1.11-4.14). Each additional year of age was associated with higher odds of moderate to severe depression (AOR, 1.03; 95% CI, 1.00-1.07). Other variables were not associated with outcomes.

**Table 2.** Association of Anxiety, Depression, and Insomnia With Sociodemographic Characteristics and War-Related Experiences

Variable^a^	Adjusted OR (95% CI)	*P* value
Model 1: moderate to severe anxiety (GAD-7)^b^		
Work in an office (vs not working)	2.75 (1.25-6.04)	.01
Residing with relatives (vs private house)	1.99 (0.98-4.02)	.06
Model 2: moderate to severe depression (PHQ-9)^c^		
Age (per 1-y increase)	1.03 (1.00-1.07)	.04
Work in an office (vs not working)	3.14 (1.33-7.42)	.009
Residing with relatives (vs private house)	2.31 (1.09-4.89)	.03
Model 3: clinically meaningful insomnia (ISI)^d^		
Work in an office (vs not working)	2.98 (1.54-5.76)	.001
Widower (vs single)	8.08 (1.33-48.99)	.02
Residing with relatives (vs private house)	2.14 (1.11-4.14)	.02

Abbreviations: GAD-7, 7-item Generalized Anxiety Disorder scale; ISI, Insomnia Severity Index; PHQ-9, 9-item Patient Health Questionnaire; OR, odds ratio.

^a^ Includes only the variables that were statistically significant (*P* < .05) or showed a strong trend (ie, *P* < .10) in the logistic regression models. Model fit statistics (Nagelkerke *R*^2^ and Hosmer-Lemeshow) test results.

^b^ Dependent variable is categorized as minimal or mild (1-9) vs moderate to severe (≥10).

^c^ Dependent variable is categorized as none or mild depression vs moderate, moderate to severe, or severe (≥10).

^d^ Dependent variable is categorized as not clinically meaningful vs clinically meaningful (≥15).

## Discussion

High psychological distress was observed among Lebanese refugees in Syria, exceeding rates reported among resettled European refugees.^1,2^ This likely reflects postdisplacement adversity, including unemployment and precarious housing. Sleep quality was poorest among participants from destroyed homes. Shared housing was associated with greater depression and anxiety than private accommodations, consistent with evidence that overcrowding exacerbates distress.^3^ Conflict exposure remained associated with depression, and injury to a distant family member was linked to worse mental health, underscoring the diffuse impact of war exposure.^4^ The elevated distress stems from the interaction of premigration trauma with structural adversities, including economic collapse and limited mental health care access. The high smoking rate suggests maladaptive coping.^5^ An urgent, 2-pronged response is needed: individual trauma-informed care and structural solutions.

Study limitations include the cross-sectional design, single-city convenience sampling, and self-reported measures, which may limit causal inference and generalizability. International agencies must bolster support for host countries.^6^ Scalable responses that integrate trauma-informed mental health care with improvements to housing and livelihoods may help mitigate the mental health risks for Lebanese refugees in Syria.

## Notes

Supplement 1. **eMethods.** Instrument Details and Statistical Analysis

Supplement 2.
Data Sharing Statement
