# Supplementary material for: Mental Health Among Lebanese Refugees in Syria
Source: JAMA Netw Open. 2026 Jan 7;9(1):e2552793. doi: 10.1001/jamanetworkopen.2025.52793 (PMC12780925; doi:10.1001/jamanetworkopen.2025.52793)
Supplement: Supplement 1. — eMethods. Instrument Details and Statistical Analysis [file jamanetwopen-e2552793-s001.pdf]

Supplementary Online Content

Al-Bitar A, Tellawi I, Janoud O, et al. Mental health among Lebanese refugees in Syria. *JAMA Netw Open.* 2025;9(1):e2552793. doi:10.1001/jamanetworkopen.2025.52793

eMethods. Instrument Details and Statistical Analysis

This supplementary material has been provided by the authors to give readers additional information about their work.

© 2026 Al-Bitar A et al. *JAMA Network Open*.

eMethods. Instrument Details and Statistical Analysis

Instrument Details:

Data were collected via structured interviews using validated scales: the GAD-7 (anxiety), PHQ-9 (depression), PSQI (sleep quality), and ISI (insomnia severity). All scales demonstrated good to excellent internal consistency (Cronbach's α: 0.75–0.89).

GAD-7: Scores were categorized into minimal (0-4), mild (5-9), moderate (10-14), or severe (15-21) anxiety per Löwe et al. (Ref).

PHQ-9: Coding and interpretation followed the protocol by Kroenke et al. (Ref).

PSQI: The global score is a sum of seven components (subjective quality, latency, duration, efficiency, disturbances, medication use, daytime dysfunction), each scored 0-3. Higher scores indicate poorer sleep quality (Ref).

ISI: Administration and scoring followed the original validation study (Ref).

Statistical Analysis:

Data were analyzed using SPSS version 26. Descriptive statistics were used for participant characteristics.
The reference category for each independent variable in the logistic regression models is detailed in the footnotes of Table 2. Multicollinearity amongst predictors was assessed using variance inflation factor (VIF) diagnostics; all VIF values were below 2.0, indicating no significant multicollinearity.

Associations were examined using χ^2 tests, ANOVA, and binary logistic regression. A p-value < 0.05 was considered statistically significant.

© 2026 Al-Bitar A et al. *JAMA Network Open*.
