# Supplementary material for: Mental Health Among Lebanese Refugees in Syria
Source: JAMA Netw Open. 2026 Jan 7;9(1):e2552793. doi: 10.1001/jamanetworkopen.2025.52793 (PMC12780925; doi:10.1001/jamanetworkopen.2025.52793)
Supplement: Supplement 2. — Data Sharing Statement [file jamanetwopen-e2552793-s002.pdf]

Data Sharing Statement

Al-Bitar. Mental Health Among Lebanese Refugees in Syria. *JAMA Netw Open*. Published January 07, 2026. doi:10.1001/jamanetworkopen.2025.52793

Data
Data available: No
